# Therapeutic Potential of Ketogenic Interventions for Autosomal-Dominant Polycystic Kidney Disease: A Systematic Review

**DOI:** 10.3390/nu17010145

**Published:** 2024-12-31

**Authors:** Donglai Li, Jessica Dawson, Jenny E. Gunton

**Affiliations:** 1 Centre for Diabetes, Obesity and Endocrinology Research (CDOER), Westmead Institute for Medical Research, Westmead, Sydney, NSW 2145, Australia; doli7548@uni.sydney.edu.au; 2 Faculty of Medicine and Health, The University of Sydney, Sydney, NSW 2066, Australia; 3 NHMRC Clinical Trials Centre, University of Sydney, Sydney, NSW 2050, Australia; jessica.dawson@sydney.edu.au; 4 Department Nutrition and Dietetics, St George Hospital, Sydney, NSW 2217, Australia; 5 Department of Diabetes and Endocrinology, Room 2040, Clinical Sciences Corridor, Westmead Hospital, Westmead, Sydney, NSW 2145, Australia

**Keywords:** autosomal-dominant polycystic kidney disease, polycystic kidney disease, ketogenic diet, ketosis, ketogenic metabolic therapy, ketone bodies

## Abstract

Background: Recent findings have highlighted that abnormal energy metabolism is a key feature of autosomal-dominant polycystic kidney disease (ADPKD). Emerging evidence suggests that nutritional ketosis could offer therapeutic benefits, including potentially slowing or even reversing disease progression. This systematic review aims to synthesise the literature on ketogenic interventions to evaluate the impact in ADPKD. Methods: A systematic search was conducted in Medline, Embase, and Scopus using relevant Medical Subject Headings (MeSH) and keywords. Studies assessing ketogenic interventions in the management of ADPKD in both human and animal models were selected for data extraction and analysis. Results: Three animal reports and six human studies were identified. Ketogenic diets (KD) significantly slowed polycystic kidney disease (PKD) progression in rats with improved renal function and reduced cystic areas. There was reduced renal fibrosis and cell proliferation. The supplementation of beta-hydroxybutyrate (BHB) in rats also reduced PKD progression in a dose-dependent manner. Human studies (*n* = 129) on KD in ADPKD reported consistent body mass index (BMI) reduction across trials, with an average weight loss of ∼4 kg. Improvements in blood pressure were also noted. Ketosis was achieved in varying degrees. Effects on kidney function (eGFR) were beneficial. Results for kidney volume were mixed but most studies were underpowered for this outcome. Lipid profiles showed increases in total cholesterol (∼1 mmol/L) and LDL cholesterol (∼0.4 mmol/L) in most studies. Safety concerns such as “keto flu” symptoms, elevated uric acid levels, and occasional kidney stones were noted. Overall feasibility and adherence to the KD were rated positively by most participants. Conclusions: Human studies are promising; however, they have been limited by small sample sizes and short durations. Larger, long-term trials are needed to assess the efficacy, adherence, and safety of ketogenic diets in people with ADPKD.

## 1. Introduction

Autosomal-dominant polycystic kidney disease (ADPKD) is the most prevalent hereditary kidney disorder, affecting approximately 1 in 1000 individuals [1]. Aung et al. reviewed the prevalence in different ethnic groups and reported that the highest prevalence is observed among Black individuals (0.073%), followed by Caucasians (0.063%), Asians (0.049%), and Hispanics (0.040%) [2]. Polycystic kidney disease (PKD) is characterised by the development of bilateral renal cysts, organ enlargement, and fibrosis, leading to a progressive decline in renal function and consequently, kidney failure in approximately 70% of patients [3,4,5,6]. In addition to manifestations arising from renal cyst growth and rupture, individuals with ADPKD are also vulnerable to extra-renal conditions such as hypertension, cerebral aneurysms, abdominal hernias, and hepatic cysts [7]. They face an increased risk of all-cause mortality, ischemic stroke, haemorrhagic stroke, and end-stage renal disease [8]. Furthermore, the estimated annual costs associated with ADPKD range from $51,970 to $68,091 USD per individual [9]. As a result, ADPKD places a substantial burden on both the healthcare system and those living with the disease.

**Figure 1:**
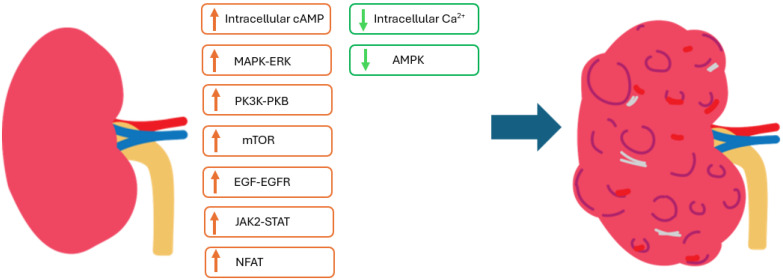
Pathological mechanisms of cyst formation in ADPKD. Cystogenesis in ADPKD is driven by a complex interplay of dysregulated signalling pathways. Mutations in polycystin 1/2 reduce calcium influx, consequently increasing intracellular cAMP, NFAT, and PI3K-PKB, which stimulate cell proliferation in cystic cells. These effects are mediated through the activation of the MAPK-ERK pathway. Additionally, renal expression of JAK2 and STAT is abnormally elevated in ADPKD, promoting excessive growth of renal cells. Elevated EGF and EGFR activity further drive cystogenesis by stimulating phosphorylation of the MAPK-ERK pathway and upregulating mTOR signalling. In addition, phosphorylation of AMPK, a negative regulator of mTOR, is reduced in cells lacking polycystin 1, further contributing to mTOR activation. Collectively, these disruptions in signalling pathways lead to uncontrolled cyst growth and expansion in ADPKD. ADPKD: autosomal-dominant polycystic kidney disease; AMPK: AMP-activated protein kinase; cAMP: cyclic adenosine monophosphate; EGF: epidermal growth factor; EGFR: epidermal growth factor receptor; JAK2: Janus kinase 2; MAPK: mitogen-activated protein kinases; mTOR: mammalian target of rapamycin; NFAT: nuclear factor of activated T cells; PI3K: phosphoinositide 3-kinase; PKB: protein kinase B; STAT: signal transducer and activator of transcription.

The primary causes of ADPKD are heterozygous mutations in the PKD genes; PKD-1 (85% of cases) and PKD-2 (15% of cases). These genes encode polycystin 1 and polycystin 2, respectively, which together form a transmembrane protein complex that plays crucial roles in regulating multiple signalling pathways involved with cellular metabolism, proliferation, differentiation, and apoptosis [10,11,12,13,14]. Decreased calcium influx, elevated cyclic adenosine monophosphate (cAMP) levels, and the abnormal activation of the mitogen-activated protein kinases (MAPK)-extracellular signal-regulated kinases (ERK) signalling pathway in renal epithelial cells are key drivers of cystogenesis [15]. Other proposed mechanisms implicated in cyst growth and expansion include dysregulated signalling pathways involving G-coupled proteins, the mammalian target of rapamycin (mTOR), phosphoinositide 3-kinase (PI3K)–protein kinase B (PKB), AMP-activated protein kinase (AMPK), Janus kinase 2 (JAK2)–signal transducer and activator of transcription (STAT), and nuclear factor of activated T cells (NFAT) [12,13,16,17,18]. Additionally, the aberrant activation of epidermal growth factor (EGF) and its receptor (EGFR) signalling has been linked to cyst expansion [19]. Figure 1 presents a simplified schematic diagram illustrating the fundamental pathological mechanisms of ADPKD.

Research has elucidated several altered metabolic processes in ADPKD cyst cells, including enhanced aerobic glycolysis, impaired fatty acid oxidation, and defective mitochondrial activity [20,21,22]. Moreover, upregulated mTOR signalling and diminished AMPK activity have been observed within these cells [20,23]. These insights highlight promising avenues for metabolic intervention. Indeed, researchers have investigated the impact of various strategies such as caloric restriction, time-restricted feeding, and the pharmacological inhibition of glycolysis and mTOR, as well as the upregulation of AMPK in ADPKD [17,18,24,25,26,27,28].

Prior to 2015, supportive measures aimed at preserving kidney function and managing complications were recommended for ADPKD [29]. These included blood pressure control, reduced salt intake, adequate hydration, and physical activity [30,31,32,33]. The sole pharmacological intervention with regulatory approval, tolvaptan, has demonstrated efficacy in slowing cyst growth [34]. However, it is not indicated for advanced-stage ADPKD and is limited by its high cost and adverse effects [35,36,37]. A systematic review and meta-analysis conducted by Griffiths et al. concluded that the other pharmacological approach, somatostatin analogues, did not benefit ADPKD patients in terms of total kidney volume (TKV) and estimated glomerular filtration rate (eGFR) [38].

Dietary recommendations for people living with ADPKD include moderating protein intake (0.55–0.60 g/kg/day to 0.75–1 g/kg/day, reducing sodium intake to less than 2300 mg/day, and maintaining adequate fluid [39,40,41]. Dietary recommendations for ADPKD are largely in line with non-ADPKD CKD, with a paucity of data evaluating the impact of diet on cyst growth or PKD progression specifically. People living with ADPKD consistently rate diet and lifestyle interventions as a high priority [42,43]. There is a need for novel dietary interventions that address the unique metabolic aspects of ADPKD such as enhanced aerobic glycolysis and impaired fatty acid oxidation [20,21,22].

Ketogenic diets (KDs) are characterized by a very low carbohydrate (typically less than 50 g per day) and high fat content [44]. This dietary regimen was originally developed to control seizures in patients with epilepsy [45] and more recently used for weight loss [46]. There is no one standard KD, with several variants used in clinical practice: the classical KD, the medium-chain triglyceride (MCT) diet, the modified Atkins diet (MAD), and the low glycaemic index treatment [47]. These variations differ in their fat-to-carbohydrate and protein ratios, generally ranging from 2:1 to 4:1 [44]. The significant reduction in carbohydrate intake leads to increased lipolysis and ketogenesis due to a low insulin-to-glucagon ratio and lower glucose availability. Adherence to the diet induces a state of ketosis, during which the body increasingly relies on ketone bodies for energy. These ketones are typically produced by the liver [48].

A transition to ketone-based metabolism has been associated with various physiological benefits, including enhanced mitochondrial efficiency, reduced oxidative stress, and the modulation of inflammatory pathways, indicating potential therapeutic effects in neurodegenerative diseases, metabolic disorders, and certain cancers [46,48,49,50,51,52,53,54,55]. Whether KD may cause cystic inhibition in ADPKD, attributed to limited carbohydrate supply, enhanced beta-oxidation, and mTOR inhibition [56,57], is of growing interest to the ADPKD community. This systematic review aims to synthesize the existing literature on the impact of ketogenic interventions in ADPKD.

## 2. Methods

A systematic review of the literature was conducted in accordance with the Preferred Reporting Items for Systematic Reviews and Meta-Analyses (PRISMA) guidelines [58]. The review is registered with the OSF Registries under the DOI: 10.17605/OSF.IO/CBXS6.

### 2.1. Eligibility Criteria

This systematic review included all published articles evaluating the use of ketogenic interventions, both dietary and supplementary approaches to achieve ketosis, in the management of ADPKD. Eligible studies were restricted to English articles and could include any type of trial, involving either human or animal subjects.

The following types of articles were excluded: letters, reviews, reports, protocols, conference abstracts, clinical practice guidelines, and editorial comments. Studies that employed fasting, intermittent fasting, dietary restriction, or other methods to achieve ketosis were not eligible. Additionally, studies focusing on diseases other than ADPKD were excluded.

### 2.2. Search Strategy

We systematically searched Medline, Embase, and Scopus databases from the earliest available date to June 2024, employing Medical Subject Headings (MeSH) and multi-purpose keywords. The search terms included “ketogenic diet” OR “ketosis” OR “ketone bodies” OR “low carbohydrate diet”, which were combined with “polycystic kidney disease” OR “autosomal dominant polycystic kidney disease” OR “renal cystic disease”. The detailed search strategies for each database are summarised in Supplementary Table S1, and were conducted on 1 June 2024.

### 2.3. Selection Process

The search results were imported into EndNote 20 (Clarivate Analytics, Philadelphia, PA, USA) and duplicates were removed. Two reviewers (D.L. and J.G.) independently screened the titles and abstracts for relevance according to the predefined inclusion and exclusion criteria. Full-text versions of potentially eligible articles were obtained for further assessment. Any disagreements were resolved through discussion and full-text evaluation.

### 2.4. Data Extraction

The information and measures were independently extracted by the same reviewers and included general study details (study type, sample size, sex, inclusion criteria, the type of ketogenic intervention, and the duration of the intervention); endpoints for animal studies (2-kidney weight to body weight ratios, serum creatinine, mitochondrial number, the quantification of kidney collagen, the quantification of fibrosis, cystic index, cyst number, cyst size, and the quantification of SMA-positive cells and Ki67-positive cells); endpoints for human studies (body weight, body mass index (BMI), blood pressure (BP), blood β-hydroxybutyrate (BHB), blood glucose, and eGFR), TKV, height-adjusted TKV (htTKV), total cholesterol (TC), low-density lipoprotein cholesterol (LDL-C), high-density lipoprotein cholesterol (HDL-C), adverse events, and adherence and feasibility to the intervention); and reported strengths and limitations of the method. Extracted data were cross-checked, and any discrepancies were resolved through consensus. We categorised the extracted data based on study characteristics, such as the type of ketogenic intervention, the duration of the intervention, and the outcomes measured. The primary endpoints for animal studies and human studies were tabulated and discussed.

### 2.5. Risk of Bias and Analysis

With the small number of studies, small numbers of subjects, and heterogeneity in study design and outcome measures, the data were not suitable for combination. Individual study results are presented in the text and tables. Risk of bias is inherently high in all of the non-randomised human studies. The single randomised placebo-controlled trial has the lowest risk of bias, noting small numbers of participants.

## 3. Results

### 3.1. Search Results

**Figure 2:**
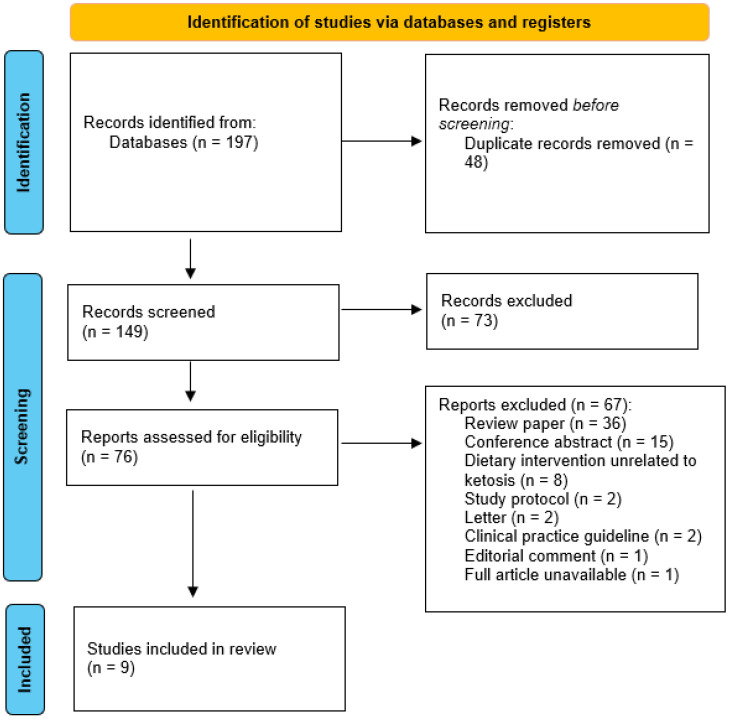
PRISMA flow diagram of article selection for the systematic review.

Database searches identified 197 records. After removing 48 duplicates, the titles and abstracts of the remaining 149 records were screened, resulting in the exclusion of 73 records. A full-text review of the remaining 76 records was conducted to assess eligibility, leading to the exclusion of 67 records. The primary reasons for exclusion were review papers (*n* = 36), conference abstracts (*n* = 15), and dietary interventions unrelated to ketosis (*n* = 8). The selection process is detailed in the PRISMA flow diagram (Figure 2).

### 3.2. Animal Studies

The only animal studies identified by the search which investigate ketogenic interventions and polycystic kidney disease were conducted by Torres and colleagues [59,60,61]. All three studies utilised the Han:SPRD rat model, which has an R823W mutation in the Anks6 gene. It closely mirrors several features of human ADPKD, including renal hyperplasia, azotaemia, and extrarenal manifestations [62]. The most recent study [61] also employed the Pkd1^RC/RC^ model and Pkd1:Cre model, featuring mutated PKD1 alleles and the kidney-specific deletion of PKD1, respectively. These models more accurately mimic human disease progression with the latter showing rapid and more aggressive cystic disease [63,64]. The characteristics of these studies are detailed in Table 1, and the summary findings are illustrated in Table 2. All animal studies reported receiving ethics approval.

#### 3.2.1. KD on Han:SPRD Rat

Juvenile rats were given ad libitum access to a severely restricted KD (~2% carbohydrates) and compared to those on a normal chow (NC) diet in Exp 1 [59]. Over five weeks, KD significantly inhibited the progression of renal cystic disease, reduced the two-kidney to body weight ratio (~30% in males and ~20% in females), and displayed an approximate 20% reduction in cystic area for both male and female animals. KD feeding improved kidney function in cystic rats, as evidenced by an increased clearance of serum creatinine by ~0.6 mg/dL (*p* < 0.0001) in male rats and ~0.3 mg/dL (*p* < 0.05) in female rats. In the control group, creatinine clearance improved by ~0.1 mg/dL (*p* < 0.05) in male rats, with no significant effect observed in female rats. Additionally, KD treatment decreased SMA-positive myofibroblasts and collagen deposition, indicating reduced fibrosis. However, KD significantly decreased Ki-67-positive cells and increased phospho-AMPK and carnitine CPT1α levels in male cystic rats only, suggesting suppressed cellular proliferation and enhanced fatty acid metabolism.

In adult rats, a 4-week KD regimen (Exp 2) induced effective ketosis [59]. There was no difference in body weight gain between wild-type and cystic animals. Notably, the total mass of polycystic kidneys remained unchanged in NC-fed rats but decreased by 35% and 20% in male and female KD-fed rats, respectively. The reduced two-kidney to body weight ratio (~20-30%) and cystic area (20%) were profound in male rats. Unlike in juvenile rats, serum creatinine levels were unaffected by KD in adult rats. However, KD significantly reduced fibrosis in male rats only, and it had no effect on myofibroblasts and the cell cycle marker Ki-67 for both male and female adult animals.

#### 3.2.2. Acute Fasting on Feline and PKD1: Nestin-Cre Models

Acute fasting was employed in various animal models to evaluate whether short-term ketosis exerts effects on PKD progression comparable to those observed with KD [59]. Given the short-term nature of these experiments, they are insufficient for evaluating the long-term renal outcomes associated with ketosis and are not included in the table below. However, they do report that after 24 h of fasting, the orthologous mouse “Pkd1:Nestin-Cre model” experienced a substantial reduction in blood glucose levels (−40%) and an increase in blood BHB concentrations (0.2 to 4.7 mM). No significant changes were observed in kidney mass or the two-kidney to body weight ratio, although apoptosis was induced in cyst-lining cells. In contrast, in a feline ADPKD model, a 72 h fasting regimen resulted in an average reduction of 15% in TKV, alongside a significant decrease in cyst size [59]. 

#### 3.2.3. BHB Supplementation on Han:SPRD Rat

Torres and colleagues investigated the effect of ketosis from high-dose BHB supplementation on PKD progression in the first study, Exp 3 [59]. Juvenile rats were treated with BHB for five weeks while being fed a high-carbohydrate diet. BHB-treated rats showed significant reductions in the two-kidney to body weight ratio (~1.3% in male and ~0.4% in female), cystic area (>30% in male and >20% in female), and collagen area (>20% in male and >10% in female). BHB also improved serum creatinine clearance by ~0.4 mg/dL in male rats and ~0.3 mg/dL in female rats, but only significantly inhibited cystic proliferation in male rats. Notably, SMA immunofluorescence stain showed an almost complete elimination of myofibroblasts.

In the second study, Torres et al. found that BHB (Exp 1) significantly reduced cystic disease in juvenile Cy/+ rats in a dose-dependent manner, evidenced by reductions in two-kidney to body weight ratios, ranging from 0.5% to 1.3%, and cystic area, with the highest BHB concentration inducing a reduction of >30% [60]. BHB also improved creatinine clearance, with the highest concentration resulting in a reduction of approximately 0.6 mg/dL, while the other two concentrations induced similar reductions of 0.3 mg/dL. Additionally, suppression in fibrosis and myofibroblast activity, and inhibited proliferation in tubule/cystic cells were observed.

The authors reported the combinations of BHB and citrate supplementation (Exp 2) significantly reduced kidney size, two-kidney to body weight ratio (~0.7–1.2%), and cystic area (~8–30%) of juvenile Cy/+ rats, with higher concentrations showing stronger effects [60]. The treatment also reduced cyst number and size in a dose-dependent manner and lowered serum creatinine levels from 3 to 5 mg/dL. Additionally, BHB/citrate significantly reduced collagen deposition and myofibroblast presence, and Ki67 assays indicated diminished proliferation in both interstitial and tubule cells. Furthermore, BHB/citrate decreased STAT3 levels dose-dependently and reduced ERK activity across all groups, unlike BHB or citrate alone. These findings suggest that combining BHB and citrate has a synergistic effect. Similar effects were found in adult rats in terms of two-kidney to body weight ratio, cystic area, and kidney function, though without significant findings for fibrosis and cell proliferation (Exp 3). Interestingly, BHB/citrate promoted mitochondrial biogenesis in kidneys of wild-type rats only.

In their most recent study, Torres and colleagues investigated the effects of BHB supplementation on adult Cy/+ rats and demonstrated significant benefits [61]. BHB reduced the two-kidney to body weight ratio in both male (~0.8%) and female (~0.3%) PKD rats. It also partially reversed cystic disease, as evidenced by a reduction in cystic area of 27% in males and 16% in females. Furthermore, BHB supplementation increased creatinine clearance by 0.6 mg/dL in male rats, although no such improvement was observed in females. Notably, serum glucose levels remained unaffected by BHB supplementation, suggesting that glucose availability did not influence disease progression. In males, BHB significantly reduced collagen deposition (~10%) and the number of Ki67-positive cells (~0.5%). However, no significant changes in SMA-1 area were observed in either sex. Additionally, BHB reduced the number of injured glomeruli compared to controls, suggesting a protective effect on glomerular health.

#### 3.2.4. BHB Supplementation on Orthologous Models

Torres and researchers utilised orthologous mouse models in their latest study to investigate the effects of BHB supplementation on PKD [61]. In the Pkd1^RC/RC^ mouse model, D-BHB-supplemented chow significantly reduced the progression of cystic disease. This included decreases in the two-kidney to body weight ratio (~0.4% in males and ~0.2% in females) and cystic area (~0.5% in males and ~0.2% in females), without affecting cyst number. Additionally, male mice exhibited a significant reduction in collagen area (~10%) and SMA-1 area (~5%), though these effects were not observed in female mice. Notably, the proliferation marker Ki67 was reduced in both male and female mice. In a more aggressive mouse model, Pkd1-Ksp:Cre, a short duration of L-BHB injections led to significant reductions in cystic area (~10%) and the two-kidney to heart weight ratio (~2.5%). Moreover, D-BHB treatment notably reduced mTORC1 signalling, with L-BHB showing a lesser extent of this effect. The study also found that elevated BHB levels induced Nrf2 expression and translocation, which subsequently enhanced fatty acid oxidation accompanied with improved PKD outcomes.

#### 3.2.5. Animal ADPKD Model Summary

In summary, a KD significantly slowed the progression of PKD in juvenile Cy/+ rats, evidenced by decreased two-kidney to body weight ratios and cystic area, and improved kidney function. However, reduced fibrosis, suppressed cell proliferation, and enhanced fatty acid metabolism were observed only in male rats [53]. In adult rats, KD induced similar effects on two-kidney to body weight ratios, but reductions in cystic area and collagen deposition were significant only in males [53]. BHB supplementation reduced PKD progression in juvenile rats in a dose-dependent manner; higher BHB concentrations led to greater reductions in two-kidney to body weight ratio, cystic area, serum creatinine, and collagen deposition, as well as suppressed fibrosis and cell proliferation [54]. The combination of BHB and citrate had similar dose-dependent effects on all clinical measures in juvenile rats, but significant benefits were observed only in adult rats for the two-kidney to body weight ratio, cystic area, and kidney function [54]. Moreover, the acute fasting experiments in mice and feline ADPKD models supported the rat studies with potential benefits of ketosis on renal outcomes [59]. Similarly, the most recent study demonstrated that BHB supplementation reduces PKD progression in adult Cy/+ rats [61]. BHB lowered the two-kidney to body weight ratio and cystic area, improved creatinine clearance in males, and reduced fibrosis and cell proliferation, with limited effects in females. Orthologous mouse models also showed similar benefits, including reduced cystic area and kidney to body weight ratios [61].

### 3.3. Human Studies

Six human studies were identified, including one randomised controlled trial (RCT), three interventional studies, a retrospective case series, and an observational cohort study [65,66,67,68,69,70]. All studies reported the impact of a ketogenic diet; there were no human studies reporting outcomes with the use of exogenous ketone supplements. The interventional studies and the randomised controlled trial included adult patients with chronic kidney disease (CKD) stage G1–3, excluding those with other chronic conditions such as diabetes and eating disorders, as well as patients on dialysis or those who had undergone kidney transplants [65,66,68,69]. All intervention and prospective studies received ethics approval. Interventional studies provided individualized interventions including meal plans and recipes [65,66,68], with two of these studies being under the guidance of a renal dietitian [65,66]. One interventional study provided the study food items to participants [69]. The retrospective case series recruited patients who self-initiated a KD and applied similar exclusion criteria [67]. The observational study included stage G4 patients and excluded those who started disease-modifying medications during follow-up and those who developed type 2 diabetes [70]. The study characteristics and findings are summarised in Table 3 and Table 4, respectively.

#### 3.3.1. Body Weight

All studies that assessed body weight and BMI reported decreases in both metrics among participants [65,66,67,68,69]. The randomized controlled trial (KETO-ADPKD) reported an average weight loss of 6.1 ± 4.0 kg in the KD group [68]. Similarly, the RESET-PKD study by Oehm et al. observed significant weight reductions in both the KD and WF groups [69]. The pilot interventional study conducted by Testa and colleagues noted that one patient lost 1 kg and another lost 4.2 kg on a relatively hypocaloric KD [65]. The Ren.Nu programme by Bruen and researchers found that 89% of participants reported weight loss, with an average weight loss of 5.6% and an average BMI reduction of 1.33 kg/m^2^ [66]. Additionally, the retrospective case series study concluded that 90% of participants on a KD experienced an average weight reduction of 8.9 kg, while the remaining 10% did not experience any weight loss [67]. It is important to note that in many cases, the weights were patient reported.

#### 3.3.2. Blood Pressure

Three studies reported a decrease in BP among participants [65,66,67]. Testa et al. found that the average decrease in BP in the three patients was statistically significant for diastolic BP (−15.7 mmHg) at the end of the study [65]. The Ren.Nu trial reported that 94% of participants self-reported improved BP [66]. The retrospective study found that 64% of participants with hypertension self-reported improved BP [67]. Two studies did not find significant changes in BP among participants, this includes the only published RCT [68,69].

#### 3.3.3. Blood Glucose and BHB

In assessing the biochemical efficacy, Testa and colleagues reported a significant decrease in glycemia 14 days after starting a KD. Ketosis was rapidly induced, with 79.5% of daily blood BHB measurements achieving the pre-set metabolic threshold of 0.3 mmol/L for all three participants [65]. Similarly, participants in the Ren.Nu program self-reported an average decrease of 16.5% in blood glucose levels and achieved ketosis and maintained it for most of the study period with blood BHB levels sustaining between 1.1 and 1.3 mM [66]. In the KETO-ADPKD trial, researchers noted that only 39% of participants met the metabolic cut-off for ketosis during all study visits [68]. The RESET-PKD study found that KD induced a significant increase in BHB levels; however, the decrease in blood glucose levels was not statistically significant [69]. Finally, the cohort study concluded that blood glucose levels were inversely associated with BHB levels [70].

#### 3.3.4. Renal Function and Kidney Volume

All studies investigated the effect of KD on kidney function. The Ren.Nu study reported an average increase in eGFR of 8.6% from baseline among the participants [66]. Similarly, studies by Strubl et al. and Cukoski et al. both concluded statistically significant increases in eGFR; however, the decrease in htTKV reported by Cukoski et al. did not reach statistical significance [67,68]. The cohort study found that doubling blood BHB levels was associated with a slower decline in eGFR by 0.33 mL/min/1.73 m^2^, but BHB levels had no association with htTKV over time [70]. The other two studies did not find any significant effect of KD on eGFR and TKV [65,69].

#### 3.3.5. Lipid Profiles

Five studies investigated the effect of KD on lipid profiles [65,66,67,68,69]. Testa et al. reported that all three participants had a significant increase in TC with a mean increase of 34 ± 13.1 mg/dL (~0.9 mmol/L). An increase in LDL-C, though not statistically significant, was observed in all participants [65]. In the retrospective case series, 10 participants reported safety concerns about increased cholesterol levels, with an average increase of 13 mg/dL in TC and 8.5 mg/dL in LDL-C, which was significantly higher in the KD group [67]. Similarly, the KETO-ADPKD trial found a significant rise in TC and LDL-C in the KD group, which aligns with results from the RESET-PKD study, where TC and LDL-C increased significantly only in the KD group [68,69]. Conversely, the Ren.Nu study reported that averages of TC and LDL-C remained unchanged among the participants [66].

For HDL-C, participants experienced a small, non-significant increase in the pilot trial [65]. This result was similar to those in the KETO-ADPKD and RESET-PKD studies [68,69]. The self-reported data in the retrospective trial showed HDL-C remained largely unchanged [67]. However, Bruen and colleagues reported that the Ren.Nu program resulted in an average HDL-C increase of 4 mg/dL over 12 weeks among participants [66].

#### 3.3.6. Safety and Adverse Events

Five studies reported safety-related adverse events [65,66,67,68,69]. Testa and researchers noted that two out of three participants experienced fatigue; of these, one also had gastrointestinal alterations, and another had muscle cramps. Notably, one participant was found to have nephrolithiasis at the end of the trial, though that was claimed to be present prior to the study [65]. The Ren.Nu study reported one case each of urinary tract infection (UTI), gout flare, and kidney stone. Researchers could not determine if these occurrences were related to dietary changes, as these conditions are common among individuals with ADPKD and the participants had histories of these issues [66]. In the retrospective study, 81% of participants reported symptoms, predominantly “keto flu” symptoms such as headaches, fatigue, nausea, foggy brain, and difficulty concentrating. Other common issues included hunger, bad breath, constipation, excessive thirst, and feeling cold. While 76% of these issues resolved over time, 12% persisted [67]. Similarly, the KETO-ADPKD study reported that 43% of participants experienced “keto flu” in the KD group. Additionally, 17% of participants had elevated uric acid levels, and 17% reported orthostatic-related symptoms. One participant developed appendicitis, and another had nephrolithiasis, both required hospitalisations [68]. In the RESET-PKD trial, in addition to “keto flu” symptoms—three cases of fatigue, two of headache, one of nausea, and one of difficulty concentrating—researchers also observed a significant increase in uric acid levels among participants [69].

#### 3.3.7. Feasibility and Adherence

Participants in the pilot study conducted by Testa and colleagues rated their satisfaction with the KD at 4/5 and their compliance at 3/5. Although they did not find it difficult to purchase, prepare, and consume the prescribed KD, adhering to a KD in social contexts proved challenging [65]. The Ren.Nu program had a completion rate of 92%, with 88% of participants rating the feasibility of the KD as four or five on a scale of one to five. While shopping, preparing, and eating the plant-based KD were relatively easy, the most challenging aspect was also participating in social events such as eating out at restaurants or friends’ homes [66]. Strubl et al. reported that 76% of participants found the KD manageable, with half stating they followed the KD every day or only skipped it a few times a month. However, 40% of participants needed breaks from the KD [67].

The RESET-PKD and KETO-ADPKD studies both established a combined feasibility endpoint, determined by metabolic efficacy through blood BHB or breath acetone measurements and participant-reported feasibility questionnaires [68,69]. In the RESET-PKD study, all patients rated the KD as feasible, and only one patient did not reach the predefined metabolic threshold for ketosis, resulting in 80% of participants meeting the combined feasibility endpoint [69]. In contrast, the KETO-ADPKD study concluded that 43% of participants achieved the combined endpoint. While 95% of patients rated the KD as feasible, only 47% met the predefined threshold for ketosis [68].

## 4. Discussion

With no existing cure for ADPKD, the pursuit of therapies that can slow or reverse the disease progression has become a primary concern for patients, community organisations, and healthcare practitioners [71]. Following the theoretic rationale and in vitro evidence [20,21,22,23], ketogenic interventions have been explored in animal models, and initial insights have been gathered from human small-scale pilot and interventional studies since 2019 [59,60,65,66,67,68,69,70]. In experimental models there are improvements in kidney function and kidney size with researchers exploring the molecular and tissue-level effects, reporting reductions in cystic area, fibrosis, and cell proliferation in KD- and BHB-treated mice PKD models, offering valuable insights for future research [59,60]. In human subjects, parameters such as body weight, BP, lipid profile, safety, and feasibility have been investigated [65,66,67,68,69].

The human studies consistently reported reductions in body weight and BP. Some studies noted a decrease in BGL and improvements in eGFR, along with mostly increased blood lipid levels.

### 4.1. Diet Adherence

Participants self-reported high adherence; however, objective measures of adherence, such as BHB levels, were lower. The treatment durations of the interventional studies, including the RCT, ranged from three weeks to three months, so are unable to address the questions of long-term adherence and impacts [65,66,68,69]. A meta-analysis of studies utilising the KD for epilepsy management revealed a combined patient compliance rate of only 45%, with reasons primarily due to intolerability from adverse effects, psychosocial factors, or the restrictiveness of the diet [72]. In a recent epilepsy study, self-catering of the KD emerged as a significant challenge, as participants found themselves overwhelmed by menu instructions and meal preparation. The authors emphasised the importance of well-structured education and support on basic principles of the KD, practical methods, and goal setting to achieve optimal compliance [73]. Notably, participants in the Ren.Nu program found meal preparation manageable and relatively easy, which might be attributed to personalised education provided on the scientific background and practical skills, as well as consistent monitoring and supervision from registered dietitians [66]. Additionally, Erkent and colleagues suggested healthcare providers should consider factors such as education level, socio-economic status, cognitive function, family support, and the presence of comorbidities when designing tailored approaches for KD implementation, to ensure long-term sustainability and lifestyle changes [73]. Whilst exogenous BHB supplementation has shown promising results in experimental models [59,70], this has yet to be evaluated in any human studies.

For the above reasons, the promising results of the animal studies using the direct supplementation of ketones are of great interest, and these will hopefully be trialled in people.

### 4.2. Diet Side-Effects

Due to the nature of KD, involving the restriction of carbohydrate intake, there is a risk of deficiencies in several micronutrients, including thiamine, folate, vitamin A, magnesium, iron, and iodine [74]. Even when consuming nutrient-dense foods, only five of the twenty-four essential micronutrient recommendations were met on the KD for the paediatric epilepsy population, with significant inadequacies observed in water-soluble vitamins [75]. Additionally, given the very low carbohydrate content of the ketogenic diet, there is a risk of a lack of dietary fibre that may adversely alter the gut microbiome and intestinal function. A reduced abundance of certain health-promoting microbiota has been observed in children with epilepsy on a KD [76], and increased gut permeability due to mucus membrane degradation by bacteria [77]. It is therefore crucial for the ketogenic diet to be administered under the guidance of a dietitian to ensure adequate micronutrient and fibre content, and supplementation may be required, particularly if being used as a long-term dietary intervention. The impact of the ketogenic diet on gut microbiome in ADPKD is yet to be evaluated.

One of the main adverse effects reported in most studies was elevated blood lipids, particularly TC and LDL-C (Table 4), which may accelerate the development of atherosclerosis and increase the risk of cardiovascular disease [78]. Consistent with a recent meta-analysis, the KD significantly increased TC and LDL-C levels in individuals of normal weight compared to controls [79]. An umbrella review by Chen et al. also concluded that the KD may lead to increases in LDL-C and TC, as well as HDL-C [80]. Conversely, a systematic review and meta-analysis found no significant differences in TC, LDL-C, HDL-C, or triglycerides when comparing the KD with a balanced diet in obese individuals [81]. Similarly, in their review, Castellana et al. found no changes in LDL-C or HDL-C, but a decrease in TC and triglycerides levels from the KD [82]. Despite these mixed results, the type and quality of foods included in the KD may play crucial roles. A review of observational and interventional studies indicated that plant-based diets are generally associated with favourable lipid profiles, including decreases in TC, LDL-C, and apolipoprotein B levels [83]. Indeed, the Ren.Nu program was the only study in which participants self-reported positive lipid outcomes, with no changes in TC or LDL-C and an increase in HDL-C levels (Table 4). This study implemented a plant-based KD with moderate amounts of eggs, fish, and dairy products [66].

Another concern for long-term KD trials is the risk of nephrolithiasis. A recent meta-analysis found that approximately 8% of individuals on the KD develop kidney stones, with most cases involving uric acid stones or mixed uric acid and calcium stones [84]. In this review, two studies reported elevated uric acid levels in participants, and three participants developed kidney stones [65,66,68,69]. However, researchers noted that hyperuricemia is commonly observed in ADPKD patients, who often exhibit low urine pH [85,86,87]. Gamage et al. evaluated the role of fluid intake in preventing nephrolithiasis, suggesting that increased water intake could be incorporated into trials to reduce the incidence of kidney stones [88]. Nevertheless, the potential link between nephrolithiasis and KD requires further investigation and should be closely monitored in future trials.

### 4.3. Future Studies

A Phase II randomized clinical trial is currently underway to evaluate the efficacy of an MAD in patients with ADPKD [89]. This trial builds on the pilot study conducted by Testa et al. in 2019 [65], expanding the sample size to 90 participants and extending the treatment period to 12 months. The MAD protocol in this study includes a plant-based diet supplemented with multivitamins and citrate. Importantly, participants with a history of dyslipidaemia or renal stones were excluded to minimise confounding factors affecting safety outcomes. While the study design does not assess the risks of micronutrient deficiency and would not be powered to examine uric acid nephrolithiasis from MAD, its findings on renal function, lipid profiles, and feasibility will be highly anticipated. A trial investigating ketone supplementation in ADPKD is currently underway [90]. This study supplies a ketone ester drink to ADPKD patients over a 56-day period. Up to 20 participants will consume up to 100 g of ketone ester daily, with the primary objectives of evaluating the intervention’s feasibility and safety, as well as its impact on kidney size and function.

### 4.4. Limitations

The limitations of this review include variability in study design, methods, and outcomes. For example, only one out of the six human studies was a randomised controlled trial. Additionally, there were differences in populations, interventions, and outcome measures. The ketogenic interventions varied, and outcome measures for some studies were largely self-reports. The interventional human studies included small sample sizes, and they were therefore not well powered for some outcomes.

## 5. Conclusions

Larger and longer-term clinical trials are necessary to fully establish the efficacy of ketone-based strategies in ADPKD. Key longer-term concerns include impacts on lipid profiles, the risk of nephrolithiasis, dietary fibre inadequacy, and the potential for micronutrient deficiencies. Ongoing support and strategies from healthcare professionals, particularly dietitians, will be essential for providing expert guidance and improving long-term adherence among ketogenic diet participants.

It appears probable that long-term diet adherence will be problematic for many patients; therefore, potential future studies in people using ketone supplements will be of great interest.

In conclusion, ketogenic interventions represent a relatively innovative approach in the treatment of ADPKD, with early findings showing positive outcomes of improved renal function and reduced kidney volumes.

## Figures and Tables

**Table 1:** Characteristics of animal study designs.

Study/ Reference	Model	Sample Sizes	Intervention	Duration
Torres et al., 2019 Exp 1 [59]	Han:SPRD juvenile rat	Cy/+ M = 8, F = 7 WT M = 13, F = 6	KD: ∼2% carbohydrate, ∼5% protein, and ∼91% fat Chow: ∼62% carbohydrate, ∼25% protein, and ∼13% fat	Postnatal D21 to D56 (5 weeks)
Torres et al., 2019 Exp 2 [59]	Han:SPRD adult rat	Cy/+ M = 12, F = 10 WT M = 10, F = 14	KD: ∼2% carbohydrate, ∼5% protein, and ∼91% fat Chow: ∼62% carbohydrate, ∼25% protein, and ∼13% fat	Postnatal D56 to D84 (4 weeks)
Torres et al., 2019 Exp 3 [59]	Han:SPRD juvenile rat	BHB: Cy/+ M = 7, F = 5 WT M = 3, F = 5 Control: Cy/+ M = 4, F = 4 WT M = 2, F = 5	BHB supplement and Na^+^/K^+^ salt in water (157 mM) Controls: Na^+^/K^+^ salt in water	Postnatal D21 to D56 (5 weeks)
Torres et al., 2024 Exp 1 [60]	Han:SPRD juvenile rat	All male WT *n* = 39 Cy/+ *n* = 39	BHB supplement as Na^+^/K^+^ salt in water at 160, 80, or 40 mM	Postnatal D21 to D56 (5 weeks)
Torres et al., 2024 Exp 2 [60]	Han:SPRD juvenile rat	Males only WT *n* = 28 Cy/+ *n* = 29	BHB + citrate: 40 mM BHB/30 mM citrate or 40 mM BHB/60 mM citrate, or 80 mM BHB/60 mM citrate Controls: normal water	Postnatal D21 to D56 (5 weeks)
Torres et al., 2024 Exp 3 [60]	Han:SPRD adult rat	Males only WT *n* = 48 Cy/+ *n* = 45	BHB + citrate: 40 mM BHB/60 mM citrate, or 80 mM BHB/30 mM citrate Controls: 1. water or 2. molar equivalent Na^+^/K^+^ solution	Postnatal D56 to D84 (4 weeks)
Torres et al., 2024 Exp 1 [61]	Adult Han:SPRD rat	BHB 12/group Controls: Cy/+ M = 10, F = 11. WT M = 8, F = 10. Controls: Cy/+ M = 8, F = 9. WT M = 7, F = 6	BHB: Solution with racemic 160 mM D/L-BHB Controls: 1. water or 2. molar equivalent Na/KCI solutions	Postnatal D56 to D84 (4 weeks)
Torres et al., 2024 Exp 2 [61]	Pkd1^RC/RC^ mouse	BHB: RC/RC M = 16, F = 16 WT M = 18, F = 10 Controls: RC/RC M = 18, F = 15 WT M = 18, F = 8	BHB: 118 g/kg (10%) D-BHB supplemented chow: 19% protein, 5% fat, 0.6% P, 0.15% Mg, 0.8% K, and 2% Na Controls: chow: ∼20% protein, ∼4.5% fat, 0.64% P, 0.22% Mg, 1.10% K, and 0.30% Na	Postnatal D21 to D105 (12 weeks)
Torres et al., 2024 Exp 3 [61]	Juvenile Pkd1-Ksp:Cre mouse	D-BHB: Ksp-Cre M = 5, F = 7 WT M = 2, F = 4. L-BHB: Ksp-Cre M = 5, F = 7 WT M = 2, F = 4 Controls: Ksp-Cre M = 10, F = 10 WT M = 5, F = 1	BHB group: 15 μmol/g daily injection with 1M D-BHB or L-BHB solution Controls: injection of equivalent volume of PBS	Postnatal D7 to D10 (3 days)

BHB, β-hydroxybutyrate; Exp, experiment; F, female; KD, ketogenic diet; M, male; PBS, phosphate buffered saline; WT, wild-type.

**Table 2:** Effect of KD or BHB supplement on clinical measures in Han:SPRD rats.

Ref/ Exp	2KD/TBW	Cystic Area	Serum Creatinine	Collagen Area	SMA	Ki67	AMPK	CPT1
[59] Exp 1	↓ M *p* < 0.003 ↓ F *p* < 0.003	↓ M *p* < 0.003 ↓ F *p* = 0.006	↓ M *p* < 0.0001 ↓ F *p* < 0.05	↓ M *p* < 0.008 ↓ F *p* < 0.04	↓ M *p* < 0.04 ↓ F *p* < 0.04	↓ M *p* < 0.04 F ns	↑ M *p* < 0.05 F ns	↑ M *p* < 0.01 F ns
[59] Exp 2	↓ M *p* < 0.002 ↓ F *p* < 0.003	↓ M *p* < 0.001 F ns	NR	↓ M *p* < 0.0001 F ns	NR	NR	NR	NR
[59] Exp 3	↓ M *p* < 0.001 ↓ F *p* < 0.01	↓ M *p* < 0.001 ↓ F *p* < 0.01	↓ M *p* < 0.001 ↓ F *p* < 0.01	↓ M *p* < 0.01 ↓ F *p* < 0.05	NR	↓ cysts M *p* < 0.01 F ns	NR	NR
[60] Exp 1	↓ *p* < 0.0001	↓ *p* = 0.0001	↓ *p* < 0.0001	↓ *p* < 0.0001	↓ *p* < 0.01	Interstitial ↓ *p* < 0.05 Tubule ↓ *p* < 0.01	NR	NR
[60] Exp 2	↓ *p* < 0.0001	↓ *p* < 0.0001	↓ *p* < 0.0001	↓ *p* < 0.0001	↓ *p* < 0.001	Interstitial ↓ *p* < 0.001 Tubule ↓ *p* < 0.0001	NR	NR
[60] Exp 3	↓ *p* < 0.0001	↓ *p* < 0.01	↓ *p* < 0.05	NR	NR	NR	NR	NR
[61] Exp 1	↓ M *p* < 0.01 ↓ F *p* = 0.036	↓ M *p* < 0.0001 ↓ F *p* < 0.0001	↓ M *p* < 0.0001 F ns	↓ M *p* = 0.04 F ns	M ns F ns	↓ M *p* < 0.0001 F ns	NR	NR
[61] Exp 2	↓ M *p* < 0.0001 ↓ F *p* < 0.0001	↓ M *p* = 0.0178 ↓ F *p* = 0.03	NR	↓ M *p* = 0.0022 F ns	↓ M *p* = 0.0043 F ns	↓ M *p* = 0.0931 ↓ F *p* = 0.0152	NR	NR
[61] Exp 3	↓ D-BHB *p* = 0.066 ↓ L-BHB *p* = 0.026	↓ D-BHB *p* = 0.089 ↓ L-BHB *p* = 0.0155	NR	NR	NR	NR	NR	NR

↓ decreased, ↑ increased, and — indicates no change; AMPK, AMP-activated protein kinase, BHB, β-hydroxybutyrate; CPT1, carnitine palmitoyltransferase I; Exp, experiment; F, female; Ki67, antigen Kiel 67; M, male; NR, not reported; ns, not significant; Ref, Reference; SMA, smooth muscle actin; 2KD/TBW, two-kidney to body weight ratio.

**Table 3:** Characteristics of human studies.

Ref	Design	M/F	Intervention/Exposure	Duration
Cukoski et al., 2023 [68]	Randomized control trial	Control: 10/9 KD: 11/12 Water Fast: 11/10	Control: ad libitum. KD (daily): 30 g CHO, protein 0.8 g/kg, NaCl < 7 g, phosphorus < 700 mg, and potassium < 4 g. Water fast: 3 consecutive days/month.	5 months
Testa et al., 2019 [65]	Intervention pilot study	2/1	MAD: 20 g CHO (5% of total calories), protein (30%), fats (65%), and multivitamin supplement	3 months
Bruen et al., 2022 [66]	Intervention study	10/14	Plant-focused KD avoiding inorganic phosphate, oxalate, and purines/urate • CHO 10–15%, protein 10–15%, and lipids 70–75% of total calories • Time-restricted (16:8 regime) • KetoCitra supplement	12 weeks
Oehm et al., 2023 [69]	Intervention study	KD: 3/2 WF: 5/0	KD group (14 days): fat/protein/CHO ratio of 10:4:1 WF group (3 days): ad libitum water and low-salt broth other days	6 to 12 weeks
Strubl et al., 2022 [67]	Retrospective case series	KD: 22/52 TRD: 16/36 CR: 2/3	• Participants followed KDIs for ~6 m • Some of the KD group additionally executed a TRD (*n* = 35) or CR (*n* = 5) • Participants in TRD mostly 16:8 regimen	N/A
Knol et al., 2024 [70]	Cohort, observational	203/318	N/A	Median 4 y

CHO, carbohydrate; CR, caloric restriction; F, female; KD, ketogenic diet; KDI, ketogenic dietary intervention; M, male; MAD, modified Atkins diet; TRD, time-restricted diet; WF, water fasting.

**Table 4:** The effects of KD on clinical measures in human studies.

Study	Δ Body Weight	BP	BGL	Blood BHB	eGFR	TC mM/L	LDL-C	HDL-C	TKV/htTKV
Cukoski et al., 2023 [68]	Con 0.27% KD −7.2% *p* = 0.007 WF −0.77%	NS	NR	↑ KD	Con −3.6% KD +13.9% *p* = 0.027 WF −9.5%	KD ↑ *p* < 0.01	KD ↑ *p* < 0.01	NS	NS
Testa et al., 2019 [65]	NS Av −1.7 kg	Sys= NS Dia ↓ *p* < 0.05	↓ 0.8 mM/L *p* = 0.004	↑	?	↑ 0.9 mM/L *p* = 0.046	NS	NS	NR
Bruen et al., 2022 [66]	Self-report ↓ 4 kg	Improved (self-report)	NS 1 mM/L	↑	NS +8.6%	NS	NS	NS	NR
Oehm et al., 2023 [69]	↓, ~2 kg	NS	↑ Control only	↑ *p* < 0.01	NS	↑ *p* = 0.015	↑ *p* = 0.029	NS	NS
Strubl et al., 2022 [67]	Self-report ↓ 9.1 kg *p* = 0.004	Self-report 132/85 to 118/76 *p* < 0.0001	NR	NR	Self-report ↑ 3.6 ml/min/1.73 m^2^ *p* <0.002	Self-report ↑ *p* < 0.01	Self-report ↑ *p* < 0.01	NS	NR
Knol et al., 2024 [70]	NR	NR	↓ *p* = 0.02	NR	Higher BHB associated with lesser decrease	NR	NR	NR	—

↓ indicates a decrease, ↑ indicates an increase, and — indicates no change; BHB, β-hydroxybutyrate; BGL, blood glucose levels; BP, blood pressure; Dia, diastolic BP; eGFR, estimated glomerular filtration rate; HDL-C, high-density lipoprotein cholesterol; htTKV, height-adjusted total kidney volume; LDL-C, low-density lipoprotein cholesterol; NR, not reported; NS, not significant; Sys, systolic BP; TC, total cholesterol; TKV, total kidney volume.

## Data Availability

The Excel file for the studies is available on request to the corresponding author.

## Supplementary Materials

The following supporting information can be downloaded at https://www.mdpi.com/article/10.3390/nu17010145/s1, Table S1: Database search strategy.

## Abbreviations

ADPKD	Autosomal-dominant polycystic kidney disease
AMPK	AMP-activated protein kinase
BHB	β-hydroxybutyrate
BMI	Body mass index
BP	Blood pressure
CKD	Chronic kidney disease
cAMP	Cyclic adenosine monophosphate
EGF	Epidermal growth factor
EGFR	Epidermal growth factor receptor
eGFR	Estimated glomerular filtration rate
ERK	Extracellular signal-regulated kinases
HDL-C	High-density lipoprotein cholesterol
JAK2	Janus Kinase 2
KD	Ketogenic diet
LDL-C	Low-density lipoprotein cholesterol
MAD	Modified Atkins diet
MAPK	Mitogen-activated protein kinases
MCT	Medium-chain triglyceride
MeSH	Medical Subject Headings
mTOR	Mammalian target of rapamycin
NC	Normal chow
NFAT	Nuclear factor of activated T cells
PI3K	Phosphoinositide 3-kinase
PKB	Protein kinase B
PKD	Polycystic kidney disease
PRISMA	Preferred Reporting Items for Systematic Reviews and Meta-Analyses
RCT	Randomised controlled trial
STAT	Signal transducer and activator of transcription
TC	Total cholesterol
TKV	Total kidney volume
UTI	Urinary tract infection
