# Supplementary material for: Therapeutic Potential of Ketogenic Interventions for Autosomal-Dominant Polycystic Kidney Disease: A Systematic Review
Source: Nutrients. 2024 Dec 31;17(1):145. doi: 10.3390/nu17010145 (PMC11723166; doi:10.3390/nu17010145)
Supplement: Supplementary file 1 [file nutrients-17-00145-s001.zip › nutrients-3366585-supplementary.pdf]

Supplementary Table S1. Search strategy 01/06/2024

Data Base	Results
**Medline**<br>Diet, Ketogenic/ OR (keto* diet* or ketosis or ketone bod* or low carbohydrat* diet* or high fat diet* or nutritional ketosis).mp. AND Polycystic Kidney Diseases/ OR (polycystic kidney disease* or PKD or autosomal dominant polycystic kidney disease* or ADPKD or renal cyst* disease*).mp.	44
**Embase**<br>exp ketone body/ or exp ketogenic diet/ or exp ketogenesis/ OR (keto* diet* or ketosis or ketone bod* or low carbohydrat* diet* or high fat diet* or nutritional ketosis).mp. AND exp kidney polycystic disease/ OR (polycystic kidney disease* or PKD or autosomal dominant polycystic kidney disease* or ADPKD or renal cyst* disease*).mp.	101
**Scopus**<br>("ketogenic diet" OR ketogen* AND diet*) AND (polycystic AND kidney AND disease)	52
